# Predominance of NDM and OXA‐48 Carbapenemase Coharboring Among Carbapenem Nonsusceptible Enterobacterales Isolates From Clinical Specimens at a Tertiary Hospital in Vietnam

**DOI:** 10.1155/ijm/5447477

**Published:** 2026-10-26

**Authors:** Loan Hong Luong, Tuyen Thanh Ha, Quynh Nguyen, Phu Thien Truong, Duy Thanh Pham, Tuan Minh Huynh, My Dung Jusselme

**Affiliations:** ^1^ Department of Microbiology and Parasitology and Gastro-Hepato Integrated Research Team (GHIRT-002.TCM2025) School of Medicine University of Medicine and Pharmacy at Ho Chi Minh City Ho Chi Minh City Vietnam hcmut.edu.vn; ^2^ Department of Medical Microbiology University Medical Center Ho Chi Minh City Ho Chi Minh City Vietnam bvdaihoc.com.vn; ^3^ Molecular Epidemiology Group Oxford University Clinical Research Unit Ho Chi Minh City Vietnam oucru.org; ^4^ Center for Tropical Medicine and Global Health Nuffield Department of Medicine University of Oxford Oxford UK ox.ac.uk; ^5^ Department of Microbiology Cho Ray Hospital Ho Chi Minh City Vietnam choray.vn; ^6^ LEESU Univ Paris Est Creteil ENPC Institut Polytechnique de Paris Creteil France u-pec.fr

**Keywords:** carbapenemase, carbapenem nonsusceptible Enterobacterales, carbapenem-resistant Enterobacterales, genotype trait, phenotype trait

## Abstract

**Background:**

Antimicrobial resistance has emerged as a growing global public health threat. The increasing of carbapenem‐resistant Enterobacterales limits treatment options. A better understanding of the phenotype and genotype distribution of carbapenemase‐producing Enterobacterales is essential to prevent, reduce and control infection. This study is aimed at characterizing the phenotypic and genotypic carbapenemase profiles of carbapenem nonsusceptible Enterobacterales isolates collected from the diverse clinical specimens at a tertiary hospital in Ho Chi Minh City.

**Methods:**

A total of 619 carbapenem nonsusceptible Enterobacterales strains were isolated from 548 clinical specimens. Species identification and antimicrobial susceptibility testing were performed using the BD Phoenix M50 system. Carbapenemase production was assessed using the modified Carbapenem Inactivation Method (mCIM). Carbapenemase‐encoding genes, including *bla*_KPC_, *bla*_NDM_, *bla*_IMP_, *bla*_OXA-23_, and *bla*_OXA-48_, in isolates with positive or inconclusive mCIM results (559 of 619) were detected by multiplex RT‐PCR.

**Results:**

The results showed that median patient age was 71 years (IQR 60–81), with 55.7% of patient being male and 28.3% admitted to the intensive care unit. *Klebsiella pneumoniae* predominated (56.5%), followed by *Klebsiella aerogenes* (14.7%) and *E. coli* (14.4%). Resistance rates were 87.7% for ertapenem and 78.7% for meropenem. mCIM positivity was high in *K. pneumoniae* (92.9%), *K. aerogenes* (93.4%), and *Serratia marcescens* (92.7%), but lower in *E. coli* (59.6%) and *E. cloacae* (40.0%). All 9 carbapenemase‐gene‐positive isolates with inconclusive mCIM carried *bla*_OXA-48_ alone. In *K. pneumoniae*, *bla*_NDM_‐*bla*_OXA-48_ co‐carriage was most common (39.3%), followed by *bla*_OXA-48_ alone (26.2%), *bla*_KPC_ alone (11.9%), and *bla*_NDM_ alone (8.3%). *E. coli* and *K. aerogenes* predominantly harbored *bla*_OXA-48_ alone (66.7% and 58.1%).

**Conclusion:**

The findings show a high burden of carbapenem nonsusceptible Enterobacterales in a Vietnamese tertiary hospital, largely driven by *K. pneumoniae* coharboring *bla*_NDM_ and *bla*_OXA-48_. Continuous molecular surveillance and strengthened antimicrobial stewardship are urgently needed to limit their dissemination in Vietnam.

## 1. Introduction

Antimicrobial resistance has emerged as one of the most pressing challenges in global public health. In 2021, an estimated 4.71 million deaths were associated with bacterial antimicrobial resistance, including 1.14 million deaths directly attributable to it, and among gram‐negative bacteria, carbapenem resistance showed the largest increase in both associated and attributable mortality of any antibiotic class between 1990 and 2021 [1]. The WHO global antibiotic resistance surveillance report 2025 further shows that resistance to carbapenems is increasing among key gram‐negative pathogens, including *Escherichia coli* and *Klebsiella pneumoniae* [2]. The Enterobacterales order comprises a diverse group of gram‐negative bacteria responsible for both community‐acquired and healthcare‐associated infections, including bloodstream infections, ventilator‐associated pneumonia, complicated urinary tract infections, and intra‐abdominal sepsis [3, 4]. The high prevalence of multidrug‐resistant (MDR) and extensively drug‐resistant (XDR) phenotypes within members of Enterobacterales has substantially narrowed therapeutic options, increasing hospitalizations and healthcare costs worldwide [1, 4]. For the treatment of severe infections caused by MDR Enterobacterales, particularly those producing extended‐spectrum *β*‐lactamases (ESBLs) or AmpC *β*‐lactamases, carbapenems are considered the last‐resort *β*‐lactam antibiotics [4, 5]. However, inappropriate antibiotic use, which accounts for approximately 30% of global consumption and is more prevalent in low‐income and middle‐income countries, is recognized as a major driver for the emergence and dissemination of carbapenem‐resistant Enterobacterales (CRE). In hospital settings, CRE infections are known to be associated with high morbidity and mortality rates because of limited availability of effective treatment options [1, 6, 7].

Among the different mechanisms of carbapenem resistance, carbapenemase production, which enables the hydrolysis of carbapenems and other *β*‐lactams, is predominant in Enterobacterales order [8–11]. According to the Ambler classification, carbapenemase enzymes can be categorized into three major classes. Class A carbapenemases are represented primarily by *K. pneumoniae* carbapenemase (KPC). Class B carbapenemases are characterized by a metallo‐*β*‐lactamase structure (MBL) and include New Delhi metallo‐*β*‐lactamase (NDM) and imipenem‐resistant Pseudomonas (IMP). Class D comprises oxacillin‐hydrolyzing carbapenemase (OXA), among which OXA‐48 is one of the most common carbapenemases and typically found in *K. pneumoniae* [5, 12]. These enzyme classes differ in hydrolytic profiles, inhibitor susceptibility, and geographic distribution, posing direct implications for treatment selection and infection control.

Epidemiological studies have shown that KPC‐producing CRE have emerged as the most prevalent carbapenemase‐producing Enterobacterales and are considered endemic in several regions worldwide. According to recent global surveillance of Enterobacterales, KPC predominates in Latin America and North America, although NDM has increased to comparable levels in the United States [10, 13, 14]. Meanwhile, NDM is the predominant carbapenemase across the Asia‐Pacific region and Africa/Middle East, followed by OXA‐48‐like enzyme, whereas OXA‐48‐like carbapenemases predominate in Europe, particularly in Turkey [13, 14]. Furthermore, coharboring multiple carbapenemases has emerged as a major global threat, especially in the Asia‐Pacific region, where NDM/OXA‐48‐like coproduction is the most common dual‐carbapenemase genotype [12–15]. Importantly, the 2024 WHO Bacterial Priority Pathogens List reaffirmed CRE as critical priority pathogens, underscoring the need for new antimicrobial agents, improved diagnostic innovations, and strengthened infection prevention and control measures [16].

Despite the escalating global burden of CRE, knowledge of the phenotypic and genotypic characteristics of carbapenemases, as well as their distribution across Enterobacterales species remains limited. A better understanding of the phenotype and genotype distribution of carbapenemase‐producing CRE is, therefore, essential for guiding targeted antimicrobial therapy and implementing infection prevention and control measures. Several methods are available for carbapenemase detection; however, the selection of an adaptable and appropriate method depends on the resources of the individual laboratory, the clinical picture and the available economic resources. Phenotypic tests require bacterial culture and identification, which take time. Among them, the modified Carbapenem Inactivation Method (mCIM) offers a practical and low‐cost assay under Vietnamese healthcare conditions [17–19]. Genotypic identification using PCR‐based assays can be performed within a few hours and rapidly determine the resistance mechanisms involved. Currently, multiplex PCR is the most commonly used molecular detection method, with many commercially available panels. However, these assays need to predefined gene targets, and not all resistance‐conferring genes are included [19, 20]. The present study aimed to investigate the phenotypic (using mCIM) and genotypic (using multiplex PCR) carbapenemase profiles of carbapenem nonsusceptible Enterobacterales isolates from hospitalized patients at a major tertiary hospital in Ho Chi Minh City, Vietnam.

## 2. Materials and Methods

### 2.1. Study Design and Clinical Specimen Collection

A cross‐sectional descriptive study was conducted from January to December 2024 at a tertiary hospital in Ho Chi Minh City, Vietnam. The study included 619 nonduplicate carbapenem nonsusceptible Enterobacterales isolates recovered from clinical specimens of 548 hospitalized patients, representing lower respiratory tract infection, urinary tract infection, bloodstream infection, skin and soft tissue infection, intra‐abdominal infection, and other infections.

### 2.2. Bacterial Identification and Carbapenem Susceptibility Testing

Enterobacterales isolates were identified using the NID panel on the BD Phoenix M50 automated system (Becton Dickinson, United States). Antimicrobial susceptibility testing for carbapenems (ertapenem, imipenem, and meropenem) was performed using the NMIC 500 panel on the same platform, and susceptibility results were interpreted per CLSI M100, 34th edition. The nonsusceptible category (encompassing both intermediate and resistant isolates) was defined as MIC ≥1 mg/L for ertapenem and ≥ 2 mg/L for imipenem and meropenem [21].

### 2.3. Phenotypic Detection of Carbapenemase Production Using the mCIM Method

All isolates exhibiting nonsusceptibility to at least one of the three carbapenems (ertapenem, imipenem, and meropenem) were evaluated for carbapenemase production using the mCIM per CLSI 2024 guidelines [21].

Specifically, a 1‐*μ*L loopful of bacterial colonies from an overnight blood agar plate was emulsified in a tube containing 2 mL trypticase soy broth and vortexed for 10–15 s. A 10‐*μ*g meropenem disk was entirely immersed in the bacterial suspension and incubated at 35^°^C ± 2°C in ambient air for 4 h ± 15 min. The meropenem disk was then placed onto a Mueller–Hinton agar plate previously inoculated with a 0.5 McFarland suspension of the meropenem‐susceptible indicator strain *E. coli* ATCC 25922 and incubated at 35^°^C ± 2°C for 18–24 h. Quality control used *K. pneumoniae* ATCC BAA‐1705 (positive control) and ATCC BAA‐1706 (negative control).

The mCIM results were interpreted based on the zone of inhibition diameter: positive (a zone of 6–15 mm, or the presence of pinpoint colonies within a 16–18 mm zone), negative (a clear zone of ≥ 19 mm), and inconclusive (a zone of 16–18 mm without pinpoint colonies, or a zone of ≥ 19 mm with pinpoint colonies present within it). Isolates with positive or inconclusive mCIM results (a total of 559 Enterobacterales isolates) were subsequently selected for genotypic analysis of carbapenemases.

### 2.4. Genotypic Detection of Carbapenemase Genes Using Multiplex Real‐Time PCR

Bacterial DNA was extracted using the boiling method. Briefly, three isolated colonies (or a 1‐*μ*L loopful) from an overnight nutrient agar culture were suspended in 100 *μ*L of nuclease‐free water. The suspension was heated at 98°C for 10 min to disrupt the bacterial cell membranes, and then placed on ice. Following brief centrifugation, the DNA‐containing supernatant was used directly as PCR template.

The study employed multiplex real‐time PCR combined with high‐resolution melting curve analysis using EvaGreen fluorescent dye (SensiFAST HRM mix, Bioline, United States) to detect five carbapenemase genes: *bla*_KPC_, *bla*_NDM_, *bla*_IMP_, *bla*_OXA-23_, and *bla*_OXA-48_. To prevent the overlapping of fluorescent signals, the target genes were divided into two separate reactions: Reaction 1 targeted *bla*_OXA-23_ and *bla*_NDM_, whereas Reaction 2 targeted *bla*_IMP_, *bla*_KPC_, and *bla*_OXA-48_. Specific primer sequences were adopted from the study by Dung et al. [20], with details described in Table S1.

The PCR amplification was conducted on a LightCycler 480 II system (Roche, United States); detailed reaction setup and thermal cycling conditions are provided in Table S2. A positive control (known carbapenemase‐producing strain) and a negative control (nuclease‐free water) were concurrently included in each PCR plate run. Gene amplification was confirmed as positive when the melting curve graph displayed a specific, known melting temperature (*Tm*) peak for each target antimicrobial resistance gene with a *Tm* peak height ≥ 3 (Table S1).

### 2.5. Statistical analysis

Data were analyzed using R software (Version 4.5.1). Qualitative variables were presented as frequencies and percentages. Associations between phenotypes (mCIM results) and genotypes (PCR results) were evaluated using the chi‐square or Fisher′s exact test when any expected cell count was less than five.

Susceptibility profiles to ertapenem, imipenem, and meropenem were dichotomized as susceptible versus nonsusceptible (intermediate and resistant results combined) and compared within each bacterial species. Differences in paired proportions across the three carbapenems were assessed using Cochran′s Q test. When the overall test was significant, pairwise comparisons between antibiotics were performed using McNemar′s test. Absolute differences in susceptibility rates (*Δ*, expressed as percentage points) between meropenem and other carbapenems were calculated to assess clinical relevance. A two‐sided *p* − value < 0.05 was considered statistically significant.

## 3. Results

### 3.1. Characteristics of Clinical Specimens

A total of 548 patients with infections caused by carbapenem nonsusceptible Enterobacterales were enrolled in the study (Table 1). The median age was 71 years (IQR, 60–81). The proportion of male patients was significantly higher than that of female patients (55.7% vs. 44.3%, *χ* ^2^ test, *p* = 0.008). Patients were admitted to a total of 20 treatment wards, with the highest proportions found in the intensive care units (28.3%) and emergency department (18.4%). Lower respiratory tract infection was the most common infection type, accounting for 50.4% of cases, followed by urinary tract infection (14.4%) and bloodstream infection (13.7%).

**Table 1:** Characteristics of patients with carbapenem nonsusceptible Enterobacterales infections.

Characteristics	*N* = 548
Age	71 (60–81)^a^
Gender	
Male	305 (55.7)
Female	243 (44.3)
Treating department	
Intensive care units	155 (28.3)
Emergency department	101 (18.4)
Department of Anesthesiology	66 (12)
Neurosurgical and Cardiovascular Intensive Care Unit	45 (8.2)
Department of Neurology	42 (7.7)
Department of Endocrinology	20 (3.6)
Department of Cardiology	17 (3.1)
Geriatrics and Palliative Care Department	16 (2.9)
Cardiovascular Surgery and Interventional Cardiology Department	16 (2.9)
Department of Pulmonology	15 (2.7)
Hepato‐Biliary‐Pancreatic Surgery Department	11 (2)
Gastrointestinal Surgery Department	10 (1.8)
Neurosurgery Department	9 (1.6)
Orthopedics and Traumatology Department	7 (1.3)
Department of Gastroenterology	7 (1.3)
Otorhinolaryngology Department	6 (1.2)
Department of Urology	2 (0.4)
Thoracic and Vascular Surgery Department	1 (0.2)
Department of Rheumatology	1 (0.2)
Obstetrics and Gynecology Department	1 (0.2)
Infection type	
Lower respiratory tract infection	276 (50.4)
Urinary tract infection	79 (14.4)
Bloodstream infection	75 (13.7)
Skin and soft tissue infection	65 (11.9)
Intra‐abdominal infection	43 (7.8)
Other infections	10 (1.8)

*Note:* Data are reported as *n* (%).

^a^Median (interquartile range Q1–Q3).

Of the 619 isolates analyzed, *K. pneumoniae* was the most prevalent species (56.5%, 350/619), predominantly recovered from lower respiratory tract infections (60.0%, 210/350), followed by bloodstream infections (12.6%, 44/350), urinary tract infections (10.9%, 38/350), and skin and soft tissue infections (8.0%, 28/350) (Figure 1). *K. aerogenes* (14.7%, 91/619), *E. coli* (14.4%, 89/619), and *S. marcescens* (6.6%, 41/619) were the next most frequently isolated species. Similar to *K. pneumoniae*, *K. aerogenes* and *S. marcescens* were predominantly recovered from lower respiratory tract infections (63.7% and 58.5%, respectively). In contrast, *E. coli* was distributed across multiple infection types, with bloodstream infections (25.8%, 23/89), intra‐abdominal infections (22.5%, 20/89), urinary tract infections (21.3%, 19/89), and skin and soft tissue infections (20.2%, 18/89) accounting for the majority of isolates.

**Figure 1:**
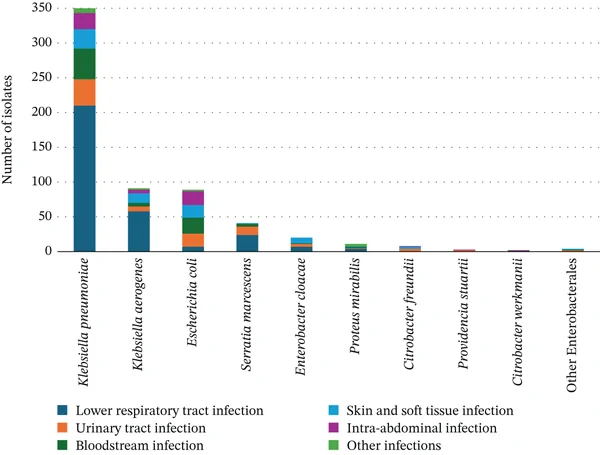
Distribution of carbapenem nonsusceptible Enterobacterales isolates by bacterial species and infection type. Each bar represents the number of isolates per species (*n* = 619), segmented by infection type. Infection types include lower respiratory tract infection, urinary tract infection, bloodstream infection, skin and soft tissue infection, intra‐abdominal infection, and other infections. Species are ordered by decreasing isolate count.

### 3.2. Carbapenem Susceptibility

The distribution of carbapenem susceptibility across species is shown in Figure 2, and detailed susceptibility profiles and statistical comparisons are presented in Table 2.

**Figure 2:**
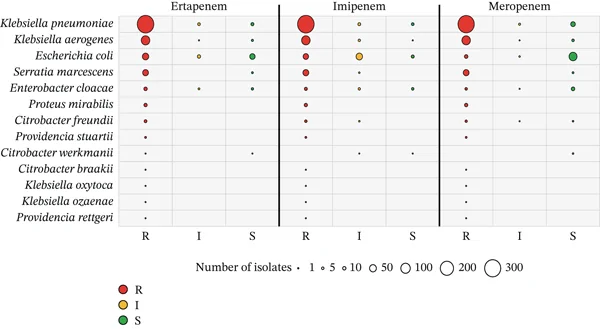
Carbapenem susceptibility patterns among carbapenem nonsusceptible Enterobacterales isolates by species and carbapenem agent. Bubble size represents the number of isolates per species in each susceptibility category (*n* = 619). Colors indicate susceptibility classifications: resistant (red), intermediate (yellow), and susceptible (green). Empty cells indicate zero isolates. Susceptibility breakpoints were interpreted per CLSI M100, 34^th^ edition (2024).

**Table 2:** Susceptibility profiles and paired comparison of ertapenem, imipenem, and meropenem by bacterial species.

Species	Type of carbapenem	Resistant *n* (%)	Intermediate *n* (%)	Susceptible *n* (%)	*p*(Cochran′s Q)	*Δ* Meropenem–Ertapenem (%)	*Δ* Meropenem–Imipenem (%)
*K. pneumoniae*	Ertapenem	334 (95.4)	7 (2.0)	9 (2.6)	< 0.001	+ 4.0	+ 4.6
	Imipenem	335 (95.7)	8 (2.3)	7 (2.0)			
	Meropenem	323 (92.3)	4 (1.1)	23 (6.6)			
*K. aerogenes*	Ertapenem	86 (94.5)	1 (1.1)	4 (4.4)	0.042	+ 2.2	+ 5.5
	Imipenem	85 (93.4)	5 (5.5)	1 (1.1)			
	Meropenem	84 (92.3)	1 (1.1)	6 (6.6)			
*E. coli*	Ertapenem	46 (51.7)	11 (12.4)	32 (36.0)	< 0.001	+ 47.1	+ 73.0
	Imipenem	33 (37.1)	47 (52.8)	9 (10.1)			
	Meropenem	13 (14.6)	2 (2.2)	74 (83.1)			
*S. marcescens*	Ertapenem	38 (92.7)	0 (0.0)	3 (7.3)	0.050	0.0	+ 7.3
	Imipenem	39 (95.1)	2 (4.9)	0 (0.0)			
	Meropenem	38 (92.7)	0 (0.0)	3 (7.3)			
*E. cloacae*	Ertapenem	12 (60.0)	3 (15.0)	5 (25.0)	0.012	+ 40.0	+ 35.0
	Imipenem	9 (45.0)	5 (25.0)	6 (30.0)			
	Meropenem	6 (30.0)	1 (5.0)	13 (65.0)			
*P. mirabilis*	Ertapenem	11 (100.0)	0 (0.0)	0 (0.0)	NA	0.0	0.0
	Imipenem	11 (100.0)	0 (0.0)	0 (0.0)			
	Meropenem	11 (100.0)	0 (0.0)	0 (0.0)			
*C. freundii*	Ertapenem	8 (100.0)	0 (0.0)	0 (0.0)	0.135	+25.0	+ 25.0
	Imipenem	6 (75.0)	2 (25.0)	0 (0.0)			
	Meropenem	5 (62.5)	1 (12.5)	2 (25.0)			
*P. stuartii*	Ertapenem	3 (100.0)	0 (0.0)	0 (0.0)	NA	0.0	0.0
	Imipenem	3 (100.0)	0 (0.0)	0 (0.0)			
	Meropenem	3 (100.0)	0 (0.0)	0 (0.0)			
*C. werkmanii*	Ertapenem	1 (50.0)	0 (0.0)	1 (50.0)	0.607	+ 50.0	+50.0
	Imipenem	0 (0.0)	1 (50.0)	1 (50.0)			
	Meropenem	0 (0.0)	0 (0.0)	2 (100.0)			
*C. braakii*	Ertapenem	1 (100.0)	0 (0.0)	0 (0.0)	NA	0.0	0.0
	Imipenem	1 (100.0)	0 (0.0)	0 (0.0)			
	Meropenem	1 (100.0)	0 (0.0)	0 (0.0)			
*K. oxytoca*	Ertapenem	1 (100.0)	0 (0.0)	0 (0.0)	NA	0.0	0.0
	Imipenem	1 (100.0)	0 (0.0)	0 (0.0)			
	Meropenem	1 (100.0)	0 (0.0)	0 (0.0)			
*K. ozaenae*	Ertapenem	1 (100.0)	0 (0.0)	0 (0.0)	NA	0.0	0.0
	Imipenem	1 (100.0)	0 (0.0)	0 (0.0)			
	Meropenem	1 (100.0)	0 (0.0)	0 (0.0)			
*P. rettgeri*	Ertapenem	1 (100.0)	0 (0.0)	0 (0.0)	NA	0.0	0.0
	Imipenem	1 (100.0)	0 (0.0)	0 (0.0)			
	Meropenem	1 (100.0)	0 (0.0)	0 (0.0)			

*Note: Δ* indicates absolute differences in susceptibility rates (percentage points) between meropenem and the other carbapenems, serving as a measure of clinical relevance. Cochran′s Q *p*‐values compare susceptibility rates across the three carbapenems within each species after dichotomization as susceptible versus nonsusceptible (intermediate and resistant combined). When the overall Cochran′s Q test was significant, pairwise comparisons between carbapenems were performed using McNemar′s test without adjustment for multiple comparisons. Statistical significance is described in the Results section.

Abbreviation: NA, not applicable due to absence of variability.

Overall, imipenem showed the highest nonsusceptibility rate (96.1%, 595/619), followed by ertapenem (91.3%, 565/619) and meropenem (80.1%, 496/619). At the species level, *K. pneumoniae* demonstrated high nonsusceptibility rates to ertapenem (97.4%, 341/350), imipenem (98.0%, 343/350), and meropenem (93.4%, 327/350), with statistically significant differences between agents (Cochran′s Q, *p* < 0.001; *Δ* < 5 percentage points); however, these absolute differences were not clinically relevant. *K. aerogenes* showed a similar pattern, with nonsusceptibility rates of 95.6% (87/91), 98.9% (90/91), and 93.4% (85/91) for ertapenem, imipenem, and meropenem, respectively (*p* = 0.042; *Δ* < 6 percentage points). *S. marcescens* exhibited high nonsusceptibility rates to ertapenem (92.7%, 38/41), imipenem (100%, 41/41), and meropenem (92.7%, 38/41), with no significant differences across carbapenems (*p* = 0.050). Similarly, *C. freundii* showed no significant differences (*p* = 0.135), with nonsusceptibility rates of 100% (8/8), 100% (8/8), and 75.0% (6/8) for ertapenem, imipenem, and meropenem, respectively. All isolates of *P. mirabilis*, *P. stuartii*, *C. braakii*, *K. oxytoca*, *K. ozaenae*, and *P. rettgeri* were nonsusceptible to all three carbapenems.

In contrast, *E. coli* demonstrated markedly higher susceptibility to meropenem (83.1%, 74/89) compared with ertapenem (36.0%, 32/89) and imipenem (10.1%, 9/89) (*Δ* = +47.1 and + 73.0 percentage points, respectively; *p* < 0.001). A similar differential susceptibility pattern was observed in *E. cloacae*, with meropenem susceptibility (65.0%, 13/20) substantially higher than ertapenem (25.0%, 5/20) and imipenem (30.0%, 6/20) (*Δ* = +40.0 and + 35.0 percentage points; *p* = 0.012), though this finding should be interpreted with caution given the small sample size (*n* = 20).

### 3.3. Phenotype of Carbapenemase by mCIM

Among the 619 carbapenem nonsusceptible isolates, the distribution of mCIM results varied by bacterial species (Figure 3). High mCIM positivity (≥ 92%) was observed in *K. pneumoniae* (92.9%, 325/350), *K. aerogenes* (93.4%, 85/91), and *S. marcescens* (92.7%, 38/41), and reached 100% in *P. mirabilis* (11/11), *C. freundii* (8/8), *P. stuartii* (3/3), *C. braakii* (1/1), *K. oxytoca* (1/1), *K. ozaenae* (1/1), and *P. rettgeri* (1/1). Lower positivity rates were observed for *E. coli* (59.6%, 53/89) and *Enterobacter cloacae* (40%, 8/20). *Citrobacter werkmanii* showed no mCIM‐positive isolates (0%, 0/2), but the sample size was limited.

**Figure 3:**
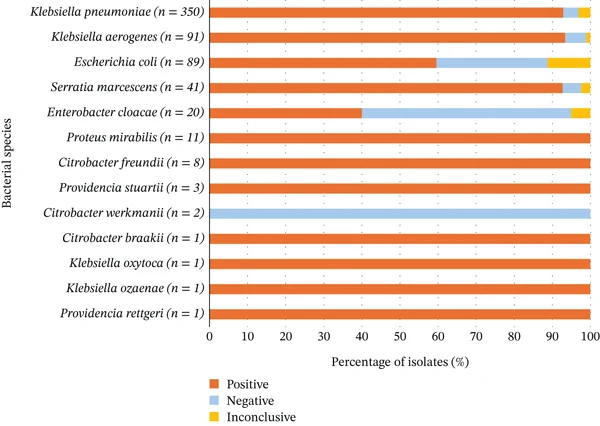
Distribution of mCIM results among 619 carbapenem nonsusceptible Enterobacterales isolates by species. Bars represent the proportion of isolates (%) within each species classified as mCIM‐positive, mCIM‐inconclusive, or mCIM‐negative. Species are ordered by decreasing number of isolates. mCIM, modified Carbapenem Inactivation Method.

### 3.4. Genotype of Carbapenemase by Multiplex Real‐Time PCR

Analysis of the distribution of carbapenemase‐encoding genes among the recovered Enterobacterales isolates revealed diversity and complexity in the carbapenem resistance mechanisms depending on the bacterial species (Figure 4). In *K. pneumoniae* (*n* = 336), isolates coharboring *bla*_NDM_‐*bla*_OXA-48_ accounted for the highest proportion (39.3%, 132/336), followed by *bla*_OXA-48_ alone (26.2%, 88/336), *bla*_KPC_ alone (11.9%, 40/336), and *bla*_NDM_ alone (8.3%, 28/336). Other genotypes included cocarriages of *bla*_KPC_‐*bla*_OXA-48_ (4.5%, 15/336), *bla*_KPC_‐*bla*_NDM_ (1.2%, 4/336), and *bla*_KPC_‐*bla*_NDM_‐*bla*_OXA-48_ (3.9%, 13/336). Sixteen *K. pneumoniae* isolates (4.8%) lacked the investigated genes.

**Figure 4:**
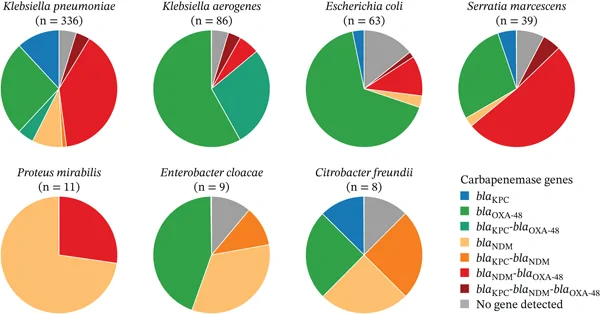
Distribution of carbapenemase genes among carbapenem nonsusceptible Enterobacterales species with ≥ 8 isolates. Data are presented as percentages of isolates per species (*n* = 559). Species with fewer than eight isolates are not shown.

In *K. aerogenes* (*n* = 86), *bla*_OXA-48_ alone was predominant (58.1%), with a notable proportion coharboring *bla*_KPC_‐*bla*_OXA-48_ (27.9%). Similarly, *bla*_OXA-48_ alone was the predominant carbapenemase genotype in *E. coli* (66.7%, 42/63), with *bla*_NDM_‐*bla*_OXA-48_ cocarriage accounting for an additional 11.1% (7/63). In *S. marcescens* (n = 39), cocarriage of *bla*_NDM_‐*bla*_OXA-48_ was the dominant genotype (51.3%), followed by *bla*_OXA-48_ alone (28.2%).

Regarding *P. mirabilis* (*n* = 11), the gene distribution exhibited distinct characteristics, with *bla*_NDM_ alone being the most common (72.7%), followed by the cocarriage of *bla*_NDM_‐*bla*_OXA-48_ (27.3%); notably, neither *bla*_KPC_ nor isolates negative for the tested genes were detected. For the less prevalent species, the majority of *E. cloacae* (*n* = 9) isolates carried *bla*_OXA-48_ alone (44.4%), whereas *C. freundii* (*n* = 8) displayed an even distribution of *bla*_OXA-48_ alone, *bla*_NDM_ alone, and the *bla*_NDM_‐*bla*_KPC_ combination (25% each). Both species contained single isolates with other profiles (e.g., *bla*_NDM_ and *bla*_KPC_ in *E. cloacae*, *bla*_KPC_ alone in *C. freundii*, and one gene‐negative isolate per species). Neither species harbored *bla*_NDM_‐*bla*_OXA-48_ cocarriage.

Notably, the group of isolates negative for the investigated genes accounted for a minor proportion across most species (ranging from 0% to 14.3%). This indicates that the carbapenem nonsusceptible phenotype among the Enterobacterales in this study is primarily driven by carbapenemase production. In addition, neither *bla*_IMP_ nor *bla*_OXA-23_ was detected in any of the isolates tested.

### 3.5. Relationship Between Phenotype and Genotype of Carbapenemase

Evaluating the performance of the phenotypic assay (mCIM) against the genotypic results (PCR) across the 559 investigated isolates revealed high concordance (Table 3). Among the 525 isolates confirmed to harbor carbapenemase‐encoding genes by PCR, the mCIM method yielded positive results for 516 isolates, achieving a positive percent agreement of 98.3%.

**Table 3:** Relationship between phenotype using mCIM and genotype using multiplex RT‐PCR carbapenemase genotypes among mCIM‐positive and mCIM‐inconclusive Enterobacterales isolates.

mCIM result	No carbapenemase gene detected (*n* = 34)	Carbapenemase gene detected (n = 525)						
		*bla*_KPC_	*bla*_NDM_	*bla*_OXA-48_	*bla*_KPC_‐*bla*_NDM_	*bla*_KPC_‐*bla*_OXA-48_	*bla*_NDM_‐*bla*_OXA-48_	*bla*_KPC_‐*bla*_NDM_‐*bla*_OXA-48_
Positive (*n* = 535)	19	45	46	189	8	39	170	19
Inconclusive (*n* = 24)	15	0	0	9	0	0	0	0

*Note:* Data are presented as number of isolates.

Abbreviation: mCIM, modified Carbapenem Inactivation Method.

Detailed analysis by genotype group demonstrated that the mCIM test possessed absolute detection capability (100%) for isolates carrying *bla*_KPC_ alone (*n* = 45), *bla*_NDM_ alone (*n* = 46), as well as all gene combinations involving *bla*_NDM_ (including *bla*_KPC_‐*bla*_NDM_, *bla*_NDM_‐*bla*_OXA-48_, and *bla*_KPC_‐*bla*_NDM_‐*bla*_OXA-48_). Conversely, inconclusive mCIM results were observed exclusively among isolates carrying *bla*_OXA-48_ alone, occurring in 4.5% (9/198) of this genotype. These 9 inconclusive isolates comprised 4 *K. pneumoniae*, 3 *E. coli*, 1 *S*. *marcescens*, and 1 *K. aerogenes*. Statistical analysis demonstrated that isolates carrying *bla*_OXA-48_ alone were significantly more likely to yield inconclusive mCIM results than those harboring any other carbapenemase genotype (*p* < 0.001, Fisher′s exact test). Of 34 PCR‐negative isolates, 19 yielded positive and 15 yielded inconclusive mCIM results.

### 3.6. Relationship Between Meropenem MICs, Carbapenemase Genes, and mCIM

Among mCIM‐positive isolates, those carrying *bla*_KPC_ alone, *bla*_NDM_ alone, or cocarriage involving *bla*_NDM_ (*bla*_NDM_‐*bla*_OXA-48_ and *bla*_NDM_‐*bla*_OXA-48_‐*bla*_KPC_) all exhibited median meropenem MICs reaching the maximum measurable threshold (> 32 mg/L). Furthermore, mCIM‐positive isolates carrying *bla*_OXA-48_ alone displayed a wide dispersion of meropenem MIC values, with a median of 32 mg/L. Notably, isolates with no target genes detected also demonstrated high meropenem MICs (median > 32 mg/L), suggesting the presence of other carbapenem resistance mechanisms beyond the investigated gene panel (Figure 5).

**Figure 5:**
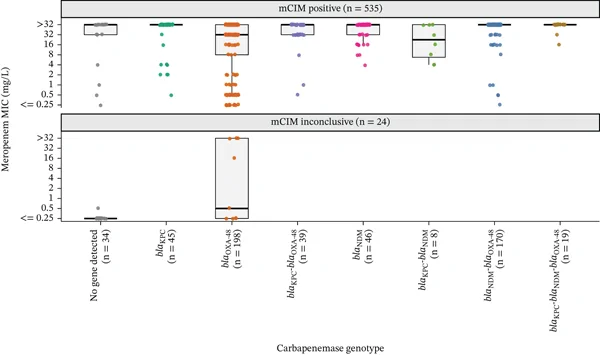
Meropenem MIC distributions among Enterobacterales isolates stratified by carbapenemase genotype and mCIM result. Box plots indicate the median (solid horizontal line) and interquartile range, overlaid with individual data points. Data are shown for 559 isolates subgrouped by mCIM result: positive (*n* = 535) and inconclusive (*n* = 24). MIC, minimum inhibitory concentration; mCIM, modified Carbapenem Inactivation Method.

Among mCIM‐inconclusive isolates, those lacking detected carbapenemase genes had low meropenem MIC levels (≤ 0.5 mg/L); whereas isolates carrying *bla*_OXA-48_ alone exhibited meropenem MIC values ranging from ≤ 0.25 mg/L (susceptible) to > 32 mg/L (resistant).

## 4. Discussion

Regarding species distribution and species‐specific carbapenemase profiles, *K. pneumoniae* was the predominant carbapenem nonsusceptible Enterobacterales species in our study, consistent with regional and global surveillance identifying it as the leading CRE pathogen in healthcare settings [13, 22]. Of note, cocarriage of *bla*_NDM_‐*bla*_OXA-48_ was the most common carbapenemase genotype among our *K. pneumoniae* isolates. This multigene cocarriage profile has been increasingly reported in Vietnam over the past decade, with progressive accumulation of *bla*_KPC_, *bla*_NDM_, and *bla*_OXA-48_ across *K. pneumoniae* lineages in both southern and northern Vietnamese hospitals [23–26]. Notably, a recent multihospital study in Ho Chi Minh City [27] identified *bla*_OXA-48_ as the dominant carbapenemase in *K. pneumoniae*, followed by *bla*_NDM_ and *bla*_KPC_, a distribution broadly reflective of the carbapenemase landscape in our study. Genomic characterization of carbapenem‐resistant *K. pneumoniae* in Vietnam has further shown that these carbapenemase genes are typically located on separate self‐transferable plasmids [28], providing a mechanistic basis for their co‐circulation. Beyond Vietnam, the cocarriage of multiple carbapenemase genes in *K. pneumoniae* has been increasingly documented across the Asia‐Pacific region [29–32], suggesting a broader regional trend rather than an institution‐specific phenomenon. This complex genotypic landscape has important implications for treatment selection. Notably, over half of *K. pneumoniae* isolates (52.7%) harbored *bla*_NDM_, either alone or in combination with other carbapenemase genes, a clinically significant proportion given that MBL‐producing infections cannot be treated with ceftazidime–avibactam alone and require more complex regimens such as ceftazidime–avibactam combined with aztreonam or cefiderocol [33–35]. These findings reinforce the essential role of molecular identification of the specific carbapenemase class in selecting the appropriate antibiotic regimen for *K. pneumoniae*.

In *K. aerogenes*, carbapenem resistance was predominantly mediated by carbapenemase production, as evidenced by high mCIM positivity (93.4%) and acquired carbapenemase gene detection (95.3%), with *bla*_OXA-48_ alone being the most common genotype. Among carbapenem‐resistant *K. aerogenes*, plasmid‐borne carbapenemases, including *bla*_KPC_, *bla*_OXA-48_, and *bla*_NDM_, have been increasingly reported in healthcare settings worldwide [36, 37]. This pattern may reflect horizontal acquisition from cocirculating Enterobacterales, particularly *K. pneumoniae*, in which *bla*_OXA-48_ was detected in nearly three‐quarters of isolates either alone or in combination with other carbapenemase genes. Supporting this hypothesis, a notable proportion of *K. aerogenes* isolates also coharbored *bla*_KPC-_ *bla*_OXA-48_, a combination also prevalent in *K. pneumoniae* in this study, suggesting interspecies horizontal transfer of carbapenemase‐encoding plasmids [38], though further genomic characterization would be needed to elucidate the underlying transmission dynamics. The carbapenemase genotype distribution in *K. aerogenes* has important implications for treatment selection. Given that the majority of *K. aerogenes* isolates lacked *bla*_NDM_ (86%), ceftazidime–avibactam monotherapy, the preferred regimen for OXA‐48‐like and KPC producers, would be applicable to most *K. aerogenes* infections in this setting.

In *E. coli*, *bla*_OXA-48_ alone was the predominant carbapenemase genotype. This finding is consistent with global molecular surveillance reporting OXA‐48‐like enzymes as the leading carbapenemase among carbapenemase‐producing *E. coli* worldwide [38, 39]. This genotypic profile provides a mechanistic explanation for the differential carbapenem susceptibility pattern observed in our *E. coli* isolates, with meropenem susceptibility substantially higher than that of ertapenem and imipenem. Such a pattern is well‐established for OXA‐48‐like producers, as these enzymes preferentially hydrolyze ertapenem while exhibiting markedly weaker activity against meropenem [38, 39]. Notably, a substantial proportion (29.2%) of *E. coli* isolates yielded negative mCIM results despite carbapenem nonsusceptibility. In this species, carbapenem resistance without detectable carbapenemase activity is well‐documented and may arise from noncarbapenemase mechanisms, particularly the combination of ESBL or plasmid‐mediated AmpC production with reduced outer membrane permeability due to OmpF/OmpC porin loss or alteration [40]. Additionally, a small subset of isolates (14.3%) yielded positive or inconclusive mCIM results without detectable carbapenemase genes by the PCR panel used, suggesting the possible presence of carbapenemase genes beyond the five targets investigated, though this could not be confirmed without in‐depth molecular characterizations.

In *S. marcescens*, *bla*_NDM_‐*bla*_OXA-48_ cocarriage was the dominant carbapenemase profile (51.3%), a finding that contrasts with the global distribution of acquired carbapenemases in this species. In large‐scale genomic analyses of *S. marcescens* from multiple countries, *bla*_KPC_ has been reported as the most prevalent acquired carbapenemase, followed by *bla*_NDM_, whereas *bla*_OXA-48_ is less common, and *bla*_NDM_‐*bla*_OXA-48_ cocarriage has been described in only isolated cases [41, 42]. Furthermore, carbapenem resistance in *S. marcescens* is more commonly attributed to noncarbapenemase mechanisms, particularly chromosomal AmpC overexpression combined with outer membrane porin loss, rather than to acquired carbapenemase genes [7]. The high frequency of *bla*_NDM_‐*bla*_OXA-48_ cocarriage observed is therefore an unusual finding for this species and may reflect local acquisition of carbapenemase‐encoding genes from cocirculating Enterobacterales within this hospital environment, though the absence of experimental data in the present study necessitates further characterization of the underlying gene transfer mechanisms.

Collectively, *bla*_OXA-48_, either alone or in combination with other carbapenemase genes, was the most widely distributed carbapenemase gene across Enterobacterales in this study, detected in 76.2% (426/559) of PCR‐tested isolates. The overlapping distribution of *bla*_OXA-48_‐containing genotypes across cocirculating species, predominating alone in *K. aerogenes* and *E. coli*, and as *bla*_NDM_ cocarriage in *K. pneumoniae* and *S. marcescens*, suggests that *K. pneumoniae*, the dominant species in this cohort, may serve as a key reservoir and disseminator of carbapenemase‐encoding plasmids within the hospital environment.

Regarding the phenotypic‐genotypic correlation of carbapenemase, the association between mCIM‐inconclusive results and *bla*_OXA-48_ reflects known biochemical differences among carbapenemase classes. Unlike NDM (Class B) and KPC (Class A), which hydrolyze carbapenems sufficiently to fully inactivate the meropenem disk within the standard 4‐h incubation, OXA‐48‐like enzymes (Class D) preferentially degrade meropenem via *β*‐lactonization rather than direct amide bond hydrolysis [43–45], resulting in incomplete disk inactivation and the characteristic pinpoint colony growth that defines an inconclusive result per CLSI 2024 criteria. This weaker hydrolytic activity explains the lower mCIM detection rate for OXA‐48 producers in our study, consistent with published literature reporting mCIM sensitivity of approximately 92%–95% for this enzyme class [17, 18, 46]. The wide meropenem MIC variability among *bla*_OXA-48_‐only isolates further suggests variable expressions of OXA‐48 enzyme and/or the presence of other molecular mechanisms. This confirms that the diagnostic challenge of OXA‐48 detection by mCIM is not a limitation of the assay itself, but rather a consequence of the inherently variable enzymatic output of this carbapenemase class [47].

Regarding the 19 isolates with positive mCIM results but no detected carbapenemase genes by PCR, this discordance does not necessarily represent mCIM false‐positivity. The mCIM phenotypic assay evaluates the total carbapenem‐inactivating capacity of the bacterial isolate, whereas the multiplex PCR panel used in this study was constrained to five specific gene targets (*bla*_KPC_, *bla*_NDM_, *bla*_IMP_, *bla*_OXA-23_, *bla*_OXA-48_). These isolates may harbor other carbapenemase genes beyond this panel, including *bla*_VIM_, *bla*_GES_, *bla*_SME_, or emerging OXA variants [48] or resistance may arise from non‐carbapenemase mechanisms, such as the combination of AmpC or ESBL hyperproduction with outer membrane impermeability due to porin loss, which can generate sufficient carbapenem‐inactivating activity to produce a positive mCIM signal. The high median meropenem MIC (> 32 mg/L) in this group confirms that these isolates are phenotypically resistant at a clinically significant level, even though the underlying resistance mechanism could not be identified by the PCR panel used in this study. This highlights the complementary strengths of phenotypic and genotypic approaches: mCIM captures overall enzymatic activity, including from uncharacterized mechanisms, whereas PCR provides targeted molecular confirmation.

This study has two key clinical implications. First, the endemic distribution of *bla*_OXA-48_ across multiple Enterobacterales species creates a specific diagnostic blind spot when mCIM is used as the sole detection method, as OXA‐48 producers are prone to yielding inconclusive rather than positive results. Reflex molecular testing for all inconclusive mCIM results is therefore warranted in settings where *bla*_OXA-48_ is endemic. Second, species‐specific carbapenemase profiles highlight the need for molecular characterization of specific carbapenemase class to select appropriate antibiotic treatment, as the distribution of *bla*_NDM_‐containing versus non‐*bla*_NDM_ genotypes varies markedly across species. *bla*_NDM_‐containing genotypes were highly prevalent in *K. pneumoniae* (52.7%) and *S. marcescens* (59%), whereas non‐*bla*_NDM_ genotypes, predominantly *bla*_OXA-48_‐based, dominated in *K. aerogenes* (86%) and *E. coli* (69.9%).

It should be noted that the present study has several strengths. It enrolled a large, consecutive sample of 619 carbapenem nonsusceptible isolates over a full calendar year, providing robust and representative data on the epidemiology and resistance mechanisms of carbapenem nonsusceptible Enterobacterales at a major tertiary hospital in southern Vietnam. The inclusion of 13 Enterobacterales species enables a broader assessment of carbapenemase distribution beyond the commonly studied *K. pneumoniae* and *E. coli*. The concurrent application of phenotypic (mCIM) and genotypic (multiplex real‐time PCR with HRM analysis) methods enabled a comprehensive paired characterization of carbapenemase production and resistance gene profiles. Furthermore, the comparative analysis of susceptibility profiles to three carbapenem agents (ertapenem, imipenem, and meropenem) across species, combined with meropenem MIC distributions stratified by carbapenemase genotype and mCIM result, provides an integrated view of the interplay between carbapenemase genotype, phenotypic carbapenemase detection, and level of carbapenem resistance.

The study also has several limitations. Although the hospital serves a large catchment area across Ho Chi Minh City and Southern Vietnam, its single‐center design may limit the generalizability of our findings. The PCR panel targeted only five carbapenemase genes commonly circulating in Vietnam, so other enzymes (e.g., *bla*_VIM_, *bla*_GES_, *bla*_SME_, and emerging *bla*_OXA_ variants) were not assessed, potentially explaining some mCIM‐positive PCR‐negative isolates. Isolates with negative mCIM results were not tested by PCR; carbapenemase gene prevalence was therefore reported among PCR‐tested isolates and may be underestimated, particularly for *bla*_OXA-48_. Finally, the absence of clonal analyses (e.g., multilocus sequence typing or whole genome sequencing) precluded identification of high‐risk lineages or outbreak‐associated strains.

## 5. Conclusion

This study provides a comprehensive characterization of carbapenem nonsusceptible Enterobacterales at a major tertiary hospital in southern Vietnam, integrating phenotypic and genotypic methods across a large, diverse isolate collection. *K. pneumoniae* emerged as the dominant pathogen with *bla*_NDM_‐*bla*_OXA-48_ cocarriage as the most prevalent genotype. The marked variation in *bla*_NDM_ prevalence across species underscores the need for molecular carbapenemase identification to guide antibiotic treatment selection. The widespread distribution of *bla*_OXA-48_ across multiple species and its specific association with inconclusive mCIM results highlight a critical diagnostic limitation of phenotypic detection alone. This study contributes to filling a critical epidemiological gap in Vietnam and Southeast Asia, providing locally grounded data on the prevalence, distribution, and diagnostic challenges of carbapenemase‐producing Enterobacterales in a healthcare setting with a high burden of carbapenem resistance.

## Author Contributions

Conceptualization: T.M.H. Methodology: P.T.T. and L.H.L. Formal analysis: Q.N. and L.H.L. Data curation: L.H.L. and T.T.H. Writing of original draft preparation: L.H.L. and D.T.P. Writing of review and editing: all authors. Supervision: T.M.H. Project administration: T.M.H. and M.D.J.

## Funding

No funding was received for this manuscript.

## Ethics Statement

The study was conducted in accordance with the principles of the Declaration of Helsinki and with relevant regulations, approved protocol, and good clinical practice. The bacterial isolates used in this study were obtained from the clinical microbiology laboratory of University Medical Center Ho Chi Minh City, where they had been isolated as part of routine diagnostic testing, and the study did not directly involve patients. The study was approved by the Institutional Review Board of University Medical Center Ho Chi Minh City (Approval Number: 18/GCN‐HDDD, dated February 16, 2024), and the requirement for informed consent to participate was waived by the Board.

## Consent

The authors have nothing to report.

## Conflicts of Interest

The authors declare no conflicts of interest.

## Supporting information


**Supporting Information** Additional supporting information can be found online in the Supporting Information section. Supporting Information File S1 contains two tables providing technical details of the multiplex real‐time PCR assay used in this study. Table S1: Primer sequences used in multiplex real‐time PCR for the detection of carbapenemase genes. Table S2: Multiplex real‐time PCR reaction setup and thermal cycling conditions.

## Data Availability

The data that support the findings of this study are available from the corresponding author upon reasonable request.
