# Supplementary material for: Predominance of NDM and OXA‐48 Carbapenemase Coharboring Among Carbapenem Nonsusceptible Enterobacterales Isolates From Clinical Specimens at a Tertiary Hospital in Vietnam
Source: Int J Microbiol. 2026 Oct 26;2026:5447477. doi: 10.1155/ijm/5447477 (PMC13698656; doi:10.1155/ijm/5447477)
Supplement: Supplementary file 1 [file IJM-2026-5447477-s001.docx]

**Predominance of NDM and OXA-48 carbapenemase co-harboring among carbapenem non-susceptible Enterobacterales isolates from clinical specimens at a tertiary hospital in Vietnam**

| **Table S1**. Primer sequences used in multiplex real-time PCR for the detection of carbapenemase genes | | | |
| --- | --- | --- | --- |
| Target gene | Primer sequence (5’ - 3’) | Amplicon size (bp) | *Tm* range (°C) |
| *bla*_OXA-48_ | F: GCG TGT ATT AGC CTT ATC GG  R: GCG GGT AAA AAT GCT TGG T | 204 | 82.0-83.0 |
| *bla*_OXA-23_ | F: ACT TGC TAT GTG GTT GCT TC  R: GAA TCA CCT GAT TAT GTC CTT | 106 | 77.8-78.8 |
| *bla*_NDM_ | F: GAC AAT ATC ACC GTT GGG AT  R: TAG TGC TCA GTG TCG GCA T | 113 | 86.3-87.3 |
| *bla*_IMP_ | F: GGT TTA AYA AAA CAA CCA CC  R: GGA ATA GAG TGG CTT AAY TCTC | 189 | 78.3-79.3 |
| *bla*_KPC_ | F: TCG TCG CGG AAC CATTC  R: ACA GTG GGA AGC GCT CCT C | 121 | 86.2-87.2 |
| F, forward; R, reverse; bp, base pairs; *Tm*, melting temperature | | |  |

| **Table S2.** Multiplex real-time PCR reaction setup and thermal cycling conditions | | | |
| --- | --- | --- | --- |
| Component | Initial concentration | Reaction 1 (µL) | Reaction 2 (µL) |
| **Reaction 1:** *bla*_OXA-23_ - *bla*_NDM_ | | | |
| Nuclease-free water | - | 3.7 | - |
| SensiFAST™ HRM mix | 2X | 10 | - |
| OXA-23-F and OXA-23-R primer mix | 20 µM | 0.8 | - |
| NDM-F and NDM-R primer mix | 20 µM | 0.5 | - |
| DNA template | - | 5 | - |
| Total (µL) |  | 20 | - |
| **Reaction 2**: *bla*_IMP_ - *bla*_KPC_ -*bla*_OXA-48_ | | | |
| Nuclease-free water | - | - | 3.5 |
| SensiFAST™ HRM mix | 2X | - | 10 |
| OXA48-F and OXA48-R primer mix | 20 µM | - | 0.5 |
| KPC-F and KPC-R primer mix | 20 µM | - | 0.5 |
| IMP-F and IMP-R primer mix | 20 µM | - | 0.5 |
| DNA template | - | - | 5 |
| Total (µL) |  | - | 20 |
| **Thermal cycling conditions (both reactions)** | | | |
| Denaturation (1X) | 95°C, 5 min | | |
| Amplification (40X) | 95°C/10 s - 60°C/30 s - 72°C/30 s | | |
| Melting curve (1X) | 95°C/2 min - 65°C/2 min - 95°C continuous | | |
| Cooling (1X) | 37°C, 30 s | | |
| HRM, High-Resolution Melting; min, minutes; s, seconds; F, forward; R, reverse | | | |
